# Blocking facial mimicry affects recognition of facial and body expressions

**DOI:** 10.1371/journal.pone.0229364

**Published:** 2020-02-20

**Authors:** Sara Borgomaneri, Corinna Bolloni, Paola Sessa, Alessio Avenanti

**Affiliations:** 1 Centro studi e ricerche in Neuroscienze Cognitive, Dipartimento di Psicologia, Alma Mater Studiorum – Università di Bologna, Campus di Cesena, Cesena, Italy; 2 IRCCS Fondazione Santa Lucia, Rome, Italy; 3 Dipartimento di Psicologia dello Sviluppo e della Socializzazione, Università degli studi di Padova, Padova, Italy; 4 Padova Neuroscience Center (PNC), Padova, Italy; 5 Centro de Investigación en Neuropsicología y Neurociencias Cognitivas, Universidad Católica del Maule, Talca, Chile; Universita degli Studi di Udine, ITALY

## Abstract

Facial mimicry is commonly defined as the tendency to imitate—at a sub-threshold level—facial expressions of other individuals. Numerous studies support a role of facial mimicry in recognizing others’ emotions. However, the underlying functional mechanism is unclear. A prominent hypothesis considers facial mimicry as based on an action-perception loop, leading to the prediction that facial mimicry should be observed only when processing others’ facial expressions. Nevertheless, previous studies have also detected facial mimicry during observation of emotional bodily expressions. An emergent alternative hypothesis is that facial mimicry overtly reflects the simulation of an “emotion”, rather than the reproduction of a specific observed motor pattern. In the present study, we tested whether blocking mimicry (“Bite”) on the lower face disrupted recognition of happy expressions conveyed by either facial or body expressions. In Experiment 1, we tested participants’ ability to identify happy, fearful and neutral expressions in the Bite condition and in two control conditions. In Experiment 2, to ensure that such a manipulation selectively affects emotion recognition, we tested participants’ ability to recognize emotional expressions, as well as the actors’ gender, under the Bite condition and a control condition. Finally, we investigated the relationship between dispositional empathy and emotion recognition under the condition of blocked mimicry. Our findings demonstrated that blocking mimicry on the lower face hindered recognition of happy facial and body expressions, while the recognition of neutral and fearful expressions was not affected by the mimicry manipulation. The mimicry manipulation did not affect the gender discrimination task. Furthermore, the impairment of happy expression recognition correlated with empathic traits. These results support the role of facial mimicry in emotion recognition and suggest that facial mimicry reflects a global sensorimotor simulation of others’ emotions rather than a muscle-specific reproduction of an observed motor expression.

## Introduction

Humans often unconsciously and unintentionally imitate or mimic others’ postures, prosody and facial expressions (e.g., [1]). *Facial mimicry* is the tendency to subtly imitate others’ facial expressions, and has been a subject of great interest among scholars during the last twenty years [2–6]. Facial mimicry reactions are, in most cases, undetectable to the human eye, and sensitive techniques are required to quantify facial muscle contractions. When electromyography (EMG) is used, these reactions are generally observed as congruent muscle responses to the observed facial expressions within one second after exposure [7–9].

The main aim of the present study was to investigate the causal involvement of facial mimicry in the recognition of facial and body emotional expressions. As detailed in the following paragraphs, such evidence would more clearly define the mechanisms underlying facial mimicry and its role in emotion recognition.

Facial mimicry has been considered a “social glue” [10], as it can generate a feeling of similarity which in turn favors prosocial behavior [11] and positive relationships [4,12]. Studies have shown a link between mimicry and the recruitment of the reward circuit, thus emphasizing the “reward value of the act of mimicking” [13]. It is, therefore, not surprising that a relationship between facial mimicry and levels of individual empathy or susceptibility to emotional contagion has been demonstrated [1,14–18], although the robustness of the relationship is still not clear (e.g., [19]). As a conspicuous line of research has strongly sustained, all these effects of facial mimicry could relate to its facilitating role in recognizing others’ expressions of emotions [20,21].

Despite the rich literature on the existence of the phenomenon and its possible functions and relevance in social contexts, the underlying psychological and neural mechanisms are less clear. One of the most influential hypotheses—also termed the “matched motor hypothesis” (e.g., [4])—takes into account an action-perception loop [3,22,23], and holds that perception and action are tightly coupled, such that re-enacting a perceived action (including a facial expression) supports its perceptual recognition [24–26]. Within this theoretical framework, facial mimicry has been conceived as linked to an internal simulation process that the observer would spontaneously put in place during the observation of others’ facial expressions and that, ultimately, would support their recognition [27–31]. In line with this proposal, neuroimaging techniques have shown that (pre)motor and somatosensory regions representing the face are active during the perception of emotional face [32–35], and neural activity in these regions correlates with the degree of facial mimicry [36–38]. Moreover, damage to, or transcranial magnetic stimulation (TMS) interference with, the face representations in motor and somatosensory areas disrupts facial mimicry and impairs the recognition and interpretation of emotional expressions [39–44] (see also [45]). Furthermore, sensorimotor simulation would in turn affect theory of mind and emotion-related regions including the amygdala and the insula [31], especially for dynamic emotional stimuli [46,47].

Although the general notion of simulation seems to be accepted by a large group of scholars, there is no agreement on how simulation processes are neurally and computationally implemented, and to what extent facial mimicry is as a fundamental “computational step” for emotion recognition [28,31]. Importantly, however, mounting evidence suggests that mimicry plays a causal role in the recognition of others’ emotional expressions [48,49]. In particular, several studies have demonstrated that altering spontaneous facial mimicry affects emotion recognition [20,48–52], modulates electrophysiological markers of face representations in visual working memory [53] and influences affective brain networks recruited during emotion perception [54,55]. Relevant to the present work is the evidence that blocking mimicry on either the lower or the upper half of the face induces specific effects on emotion recognition. For example, Oberman and colleagues [48] asked participants to bite a pen to block mimicry on the lower half of the observer’s face. These authors found that the bite manipulation specifically impaired the ability to recognize happy faces and, to some extent, disgusted faces, whereas it did not affect recognition of fearful or sad expressions. Ponari and colleagues [49] replicated the findings for happiness and disgust, and further demonstrated that blocking mimicry of the upper face resulted in poorer recognition of anger. No effects were reported for surprise and sadness, whereas blocking mimicry of both the upper and the lower face reduced recognition of fear. Two further studies showed that blocking mimicry on the lower face reduced the ability to distinguish authentic and false smiling expressions [56,57] (see also [51]). Taken together, these studies show a good correspondence between blocking specific facial muscles (e.g., orofacial muscles which are involved in smiling) and the recognition of selected facial expressions involving the same muscles (e.g., happiness), although results are not entirely consistent for all emotions (e.g., fear).

Another recent line of evidence suggests that the relationship between perceived and “enacted” sensorimotor patterns is not of the one-to-one type. It is known that the tendency to mimic others’ expressions is not limited to the observation of facial expressions. Indeed, studies indicate that seeing emotional bodily expressions also induces sensorimotor activity in the observer’s motor and somatosensory areas [58–65], and TMS interference with sensorimotor areas affects recognition of emotional body postures [59,66–68]. Interestingly, when seeing an emotional body, even when no face is observable, people tend to show motor responses in facial muscles, and these responses appear emotionally congruent with the observed body expression [69–72]; conversely, viewing fearful and angry facial expressions elicited muscular responses not only at the level of the face, but also at the level of the arm muscles involved in defensive postures [73]. Yet, the nature of these muscular responses is unclear. On the one hand, they may reflect epiphenomenal activity, i.e., a simple by-product of previously established associations between making and perceiving emotional expressions with no functional relevance to emotion recognition. On the other hand, they could causally contribute to emotion recognition as they do during observation of facial emotional expressions.

Given these premises, we, first of all, wanted to test whether blocking facial mimicry could interfere with the recognition of facial emotional expressions and, furthermore, whether this interference could extend to the recognition of bodily expressions. This kind of evidence would embrace a model of simulation in which facial sensorimotor signals impact visual recognition of emotional expressions because of their modulatory effect on a covert simulation of a well-structured motor program (i.e., one that includes more than a facial component) linked with a specific emotion.

In two behavioral experiments, we tested whether blocking mimicry on the lower face (i.e., during a “Bite” condition in which participants followed the classical biting-a-pen procedure) disrupted recognition of happy expressions conveyed by either facial or body expressions [48,49]. Specifically, in Experiment 1 we tested participants’ ability to identify happy, fearful and neutral expressions in the Bite condition and in two control conditions requiring the participant to hold a pen with the lips (“Lip”) or to keep the face relaxed (“Rest”). In Experiment 2, we tested participants’ ability to recognize the happy and fearful expressions of female/male models. Moreover, to ensure that such an effect is specific to emotion recognition, we tested whether any impairment is found when participants are asked to discriminate the gender of the models in the Bite and Lip conditions. Importantly, in both experiments, participants performed the tasks with both facial and body stimuli. Based on prior work [48,49], we expected that biting a pen would consistently reduce recognition accuracy for happy facial expressions. If the sensorimotor simulation underpinning facial mimicry consists of a low-level, muscle-specific imitation that provides a body-part specific signal aiding visual processing, we expect that the Bite condition should disrupt recognition of happy faces but not happy bodies. On the other hand, if facial mimicry is a “piece” of a more abstract and global sensorimotor simulation of observed expressions, then we expect that the “Bite” condition should reduce accuracy for happy expressions conveyed by both faces and bodies.

A final aim of the study was to investigate the relationship between dispositional empathy and the effect of blocking mimicry on emotion recognition. Indeed, facial mimicry is considered a relatively automatic process and yet it has multiple motivational and contextual moderators, including individual characteristics like empathic tendencies [1,4,31]. In particular, inter-individual differences in emotional empathy (rather than cognitive empathy) were found to predict the magnitude of spontaneous facial mimicry when observing facial expressions [14,16,18,74]. Therefore, in this study, we asked participants to fill out the Interpersonal Reactivity Index (IRI) questionnaire [75] which separately assesses emotional and cognitive aspects of empathy dispositions. Based on studies of facial mimicry (e.g., [1]), we expected that emotional empathy would predict the effect of blocking mimicry on emotion recognition.

## Materials and methods

### Participants

Seventy-nine healthy participants took part in the study. Twenty-four participants (10 men, mean age ± SD: 25.8y ± 2.4) were assigned to Experiment 1, and another 24 participants (11 men, 23.3y ± 3) were assigned to Experiment 2. Moreover, 20 participants (9 men, 26.2y ± 3.0) and 11 participants (4 men, 24.7y ± 1.2) took part in pilot studies 1 and 2, respectively. All participants were naïve to the purpose of the experiment and gave their written informed consent before being tested. The experimental procedures were approved by the University of Bologna Bioethics Committee and were carried out in accordance with the 1964 Declaration of Helsinki.

### Stimuli

**Fig 1:**
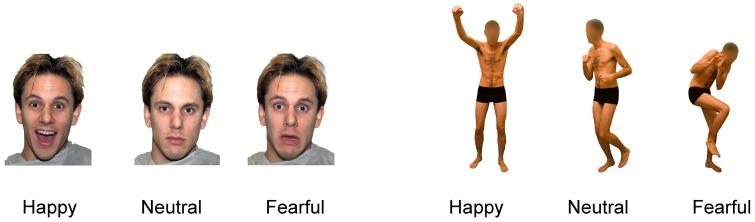
Examples of face and body stimuli in Experiment 1. Stimuli included happy, neutral and fearful expressions of the face or the body.

In Experiment 1, stimuli consisted of photographs (1000 x 1500 pixels) depicting either the face or the body of male actors showing happy, neutral or fearful expressions (Fig 1). We selected body stimuli from a validated database where facial information was removed from the body images by blurring [58,76]. Moreover, we adapted facial stimuli from the Nimstim database [77]. From an initial pool of 372 stimuli, we selected a total of 138 pictures (23 pictures for each combination of face/body medium and happy/neutral/fearful expression) based on the results of a first pilot study whose aim was to identify two sets of facial and body expressions matched for emotional intensity. We selected happy and fearful stimuli with relatively high, but not extreme, ratings, to ensure visual recognition was not trivial (see S1 Text and S1 Table), as sensorimotor simulation is thought to contribute to visual perception particularly when stimuli are relatively subtle [29,31,78,79].

In Experiment 2, face and body stimuli consisted of photographs of male and female actors from the same databases used in Experiment 1 [58,76,77,80]. Each face was cropped in an elliptical shape to exclude hair, ears and necks, thus eliminating their potential influence on gender or emotion recognition [81–84]. Similarly, the chest of each body was occluded using a black strip. These changes were introduced to avoid that a single diagnostic feature (e.g., presence of long hair or female breasts) could make gender recognition too easy.

**Fig 2:**
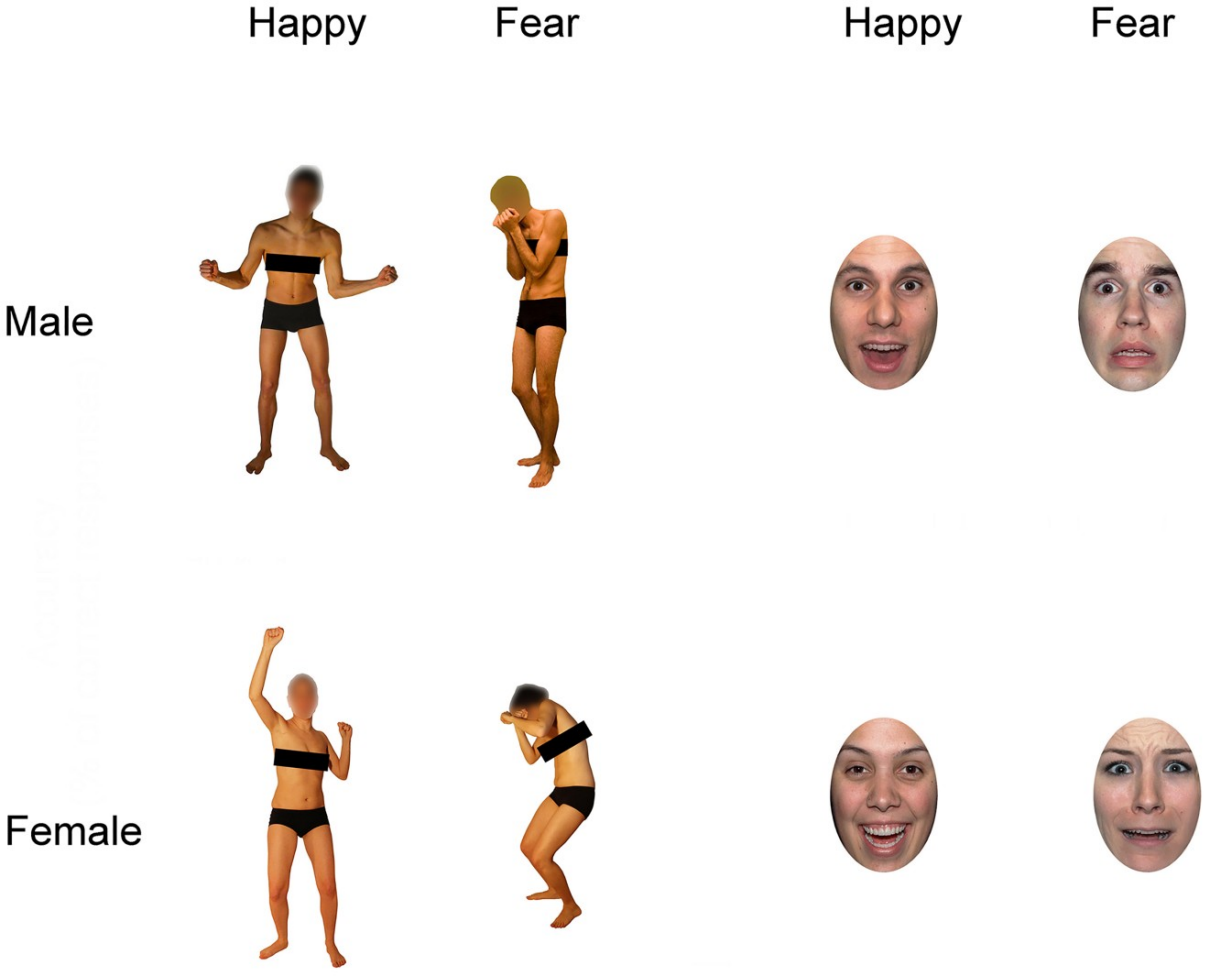
Examples of face and body stimuli in Experiment 2. Stimuli included male and female actors showing happy and fearful expressions of the face or the body.

From an initial pool of 278 stimuli, we selected a total of 80 pictures (10 pictures for each combination of face/body medium, happy/fearful expression and female/male gender) based on the results of a second pilot study. The aim of the pilot study was to choose a pool of stimuli with models whose emotional expression and gender could be recognized with high (>80%) and comparable accuracy (see S1 Text and S2 Table). Then each of the 80 pictures was mirror-reversed, resulting in a total of 160 stimuli (80 faces and 80 bodies; Fig 2).

### Procedure and experimental design

All experiments were programmed using MATLAB software to control picture presentation and to record participants’ responses. In Experiment 1, participants took part in two sessions whose order was randomized across participants. In one session they were presented with facial stimuli, whereas in the other session they were presented with body stimuli. In both sessions, participants performed an emotion recognition task during which they were asked to identify the emotion conveyed by the model’s facial/bodily expression. The expression could be happy, neutral or fearful. For each face/body medium, there were three blocks of 138 stimuli each (3 emotional expressions x 23 stimuli x 2 repetitions). In each block, participants performed the emotion discrimination task under three different facial manipulation conditions, in which they were requested to: (1) sit and relax their face (“Rest”); (2) place a pen in their mouth horizontally and hold on to it using their teeth while not allowing their lips to touch the pen (“Bite”); or (3) place a pen in their mouth horizontally and hold onto it using their lips while not allowing their teeth to touch the pen (“Lip”). Thus, the experiment consisted of 828 trials with 46 trials in each cell, constituting a 2 (Medium: face, body) x 3 (Facial manipulation: Rest, Bite, Lip) x 3 (Expression: happy, neutral, fearful) factorial design. The order of the blocks and stimuli within each block were randomized.

In Experiment 2, participants underwent two consecutive sessions, one with facial stimuli and the other with body stimuli. In each session, participants performed two different tasks, an emotion recognition task and a gender recognition task. In the emotion recognition task, participants were presented with a facial/body expression of happiness or fear and were asked to identify the emotion. In the gender discrimination task, participants were presented with the same set of stimuli used for the emotion recognition task and were asked to identify the gender of the model. For each face/body session and task, there were four experimental blocks of 160 stimuli each. Each block was divided into two halves, during which participants underwent the same “Bite” and “Lip” conditions used in Experiment 1 while they were presented with happy/fearful expressions of female and male models in a randomized order (2 emotional expressions x 2 genders x 20 stimuli x 2 facial manipulations). The experiment consisted of 640 trials, following a 2 (Medium: face, body) x 2 (Task: emotion recognition, gender recognition) x 2 (Facial manipulation: Bite, Lip) x 2 (Expression: happy, fearful) x 2 (Gender: female, male) factorial design. For data analysis, this design was simplified by collapsing data across the factor that was not of theoretical interest—Gender—and by considering the temporally contiguous conditions within each block (i.e., each combination of the factors Facial manipulation and Expression) as a single factor with 4 levels. In this way, we reached a sufficient number of 40 trials per cell for a 2 (Medium: face, body) x 2 (Task: emotion recognition, gender recognition) x 4 (Condition: Bite-happy, Bite-fearful, Lip-happy, Lip-fearful) factorial design.

**Fig 3:**
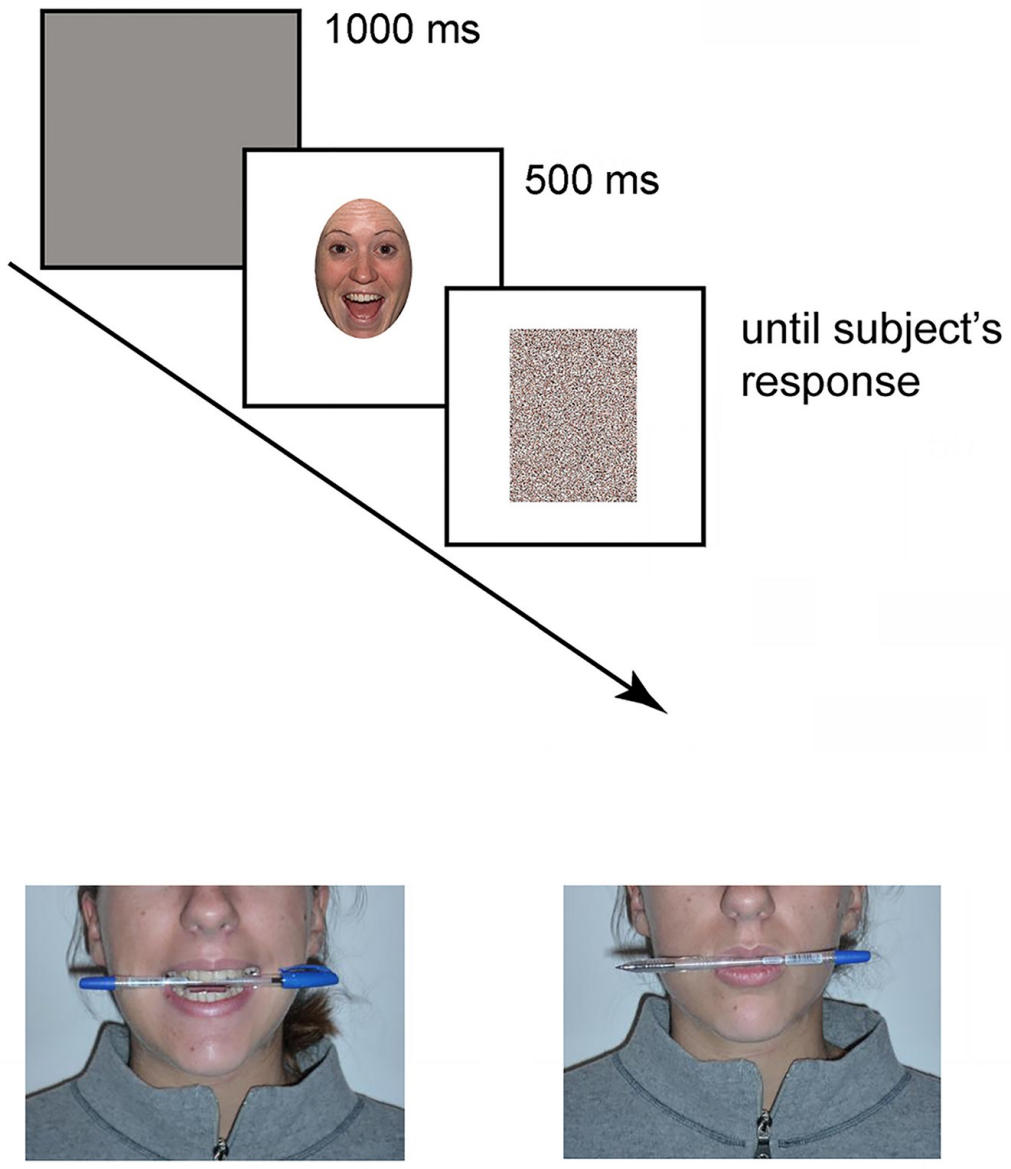
Trial example and facial manipulation.

In both experiments, the trial sequence was as follows: a gray screen (1-s duration) indicated the beginning of the trial, followed by the test picture presented at the center of the screen for 500 ms (Fig 3). The picture was followed by a random-dot mask (obtained by scrambling the corresponding sample stimulus by means of a custom-made image segmentation software) lasting until the participant’s response. Participants were instructed to provide their answer by button press and to respond as quickly and accurately as possible. After the response, the screen appeared black for 2 s in the inter-trial interval.

### Dispositional empathy

At the end of the experiment, participants were asked to complete the Interpersonal Reactivity Index (IRI) [75], a 28-item self-report questionnaire assessing dispositional empathy. The IRI consists of four scales. Two scales assess emotional empathy: the Empathic Concern (EC) scale, which assesses the tendency to feel sympathy and compassion for others in need, and the Personal Distress (PD) scale, which assesses the extent to which an individual feels distress in emotionally distressing interpersonal contexts. EC and PD correspond to the notions of other-oriented sympathy responses and self-oriented emotional distress, respectively. The other two subscales assess cognitive empathy: the Perspective Taking (PT) scale, which assesses the tendency to spontaneously imagine and assume the cognitive perspective of another person, and the Fantasy scale (FS), which assesses the tendency to project oneself into the place of fictional characters in books and movies.

### Data analysis

Accuracy (% of correct responses) and response times (RTs) were processed off-line. RTs were extracted for each trial associated with a correct response (90% and 93% in the two experiments, respectively) and RTs longer than 3 s were removed from the analysis (<1% in both experiments) before computing the median RT value. To reduce skewness, median RTs were then log-transformed for data analysis. In Experiment 1, accuracy and RTs were analyzed with a 3-way repeated measures ANOVA with Medium (2 levels: Face and Body), Facial manipulation (3 levels: Rest, Bite and Lip) and Expression (3 levels: Happy, Neutral and Fear) as within-subjects factors. In Experiment 2, despite the original experimental design including 5 factors, to reach a sufficient number of trials per cell and simplify the analysis, we submitted accuracy and RTs to a 3-way repeated measures ANOVA with Medium (2 levels: Face and Body), Task (2 levels: Emotion and Gender discrimination) and Condition (4 levels: Bite-happy, Bite-fearful, Lip-happy, Lip-fearful) as within-subjects factors.

A further mixed factors ANOVA with the between-subjects factor Experiment (2 levels: Experiment 1 and Experiment 2) and the within-subjects factors Medium (2 levels: Face and Body), Facial manipulation (2 levels: Bite and Lip) and Expression (2 levels: Happy and Fear) was carried out to compare emotion recognition accuracy between the two experiments. In all the ANOVAs, post hoc comparisons were performed using Newman–Keuls tests to correct for multiple comparisons. Partial eta squared (*η*_*p*_^*2*^) was computed as a measure of effect size for the main effects and interactions, whereas repeated measures Cohen’s *d* was computed for post hoc comparisons [85,86].

To test whether inter-individual differences in empathy influenced the effect observed in the ANOVAs (i.e., the drop in emotion recognition when biting a pen), further analyses were performed. An index reflecting the drop in accuracy in the Bite condition was computed as the mean emotion recognition accuracy for happy expressions in the bite condition minus the mean emotion recognition accuracy in the control conditions (lip-happy, bite-fear and lip-fear). This index was entered as a dependent variable into a general regression model with each subscale of the IRI as a continuous predictor and the factor Experiment as a categorical predictor. The model tested the influence of each predictor and 2-way interaction between the factor Experiment and each of the IRI subscales, to test whether similar relationships were found across the two experiments. Indices of effect size used for regression and correlation analyses included *η*_*p*_^*2*^ and Cohen’s *f*^*2*^ [85,86].

## Results

### Experiment 1

**Fig 4:**
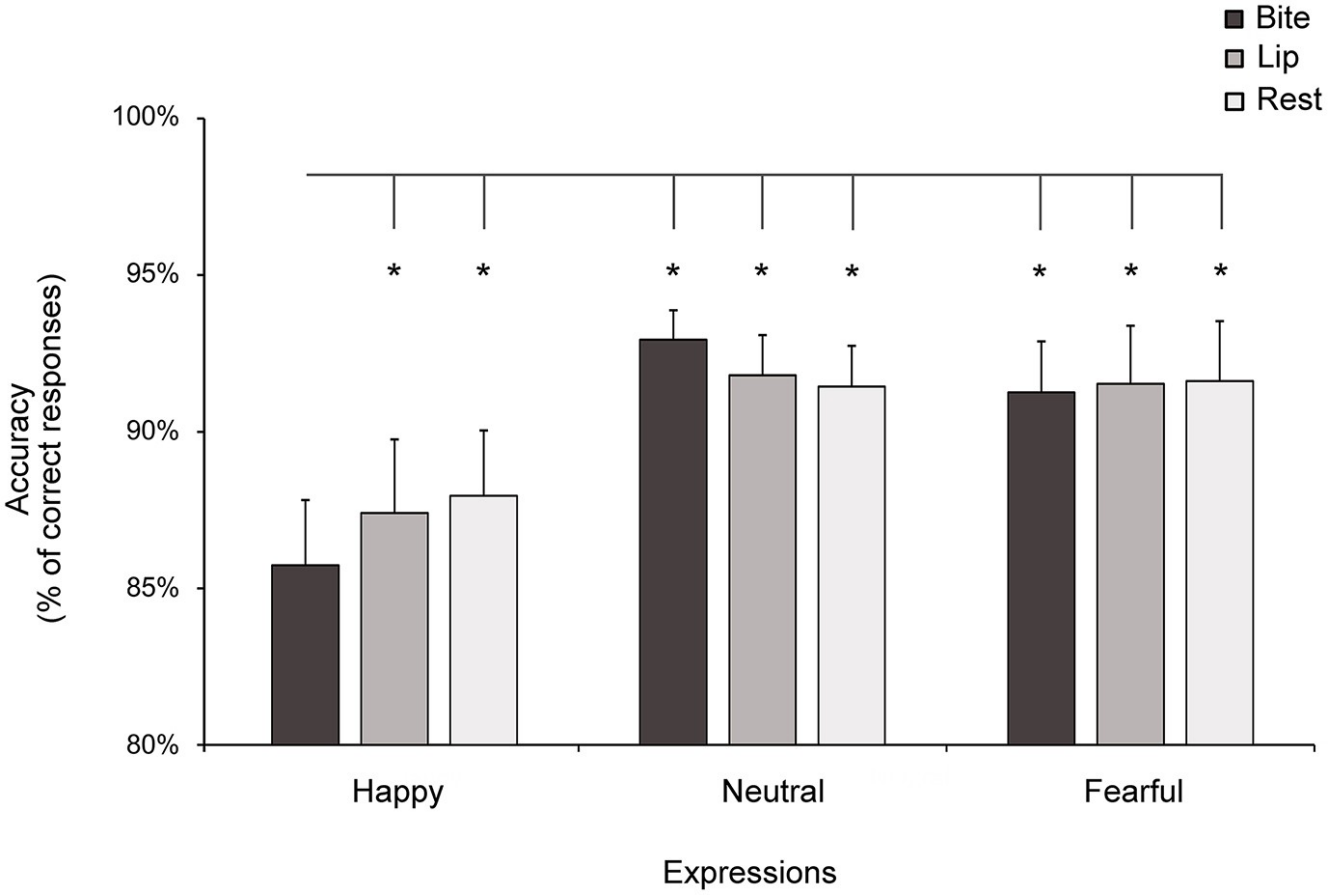
Mean accuracy in Experiment 1. When biting a pen, participants’ ability to recognize happy expressions was decreased relative to all the other conditions. Asterisks indicate significant comparisons. Error bars indicate s.e.m.

The Medium x Facial Manipulation x Expression ANOVA carried out on accuracy data showed a significant main effect of the factor Medium (*F*_1,23_ = 16.27; *p* < 0.001; *η*_*p*_^*2*^ = 0.41), indicating that participants discriminated emotional expressions with greater accuracy when emotions were conveyed by facial expressions (mean accuracy ± SD: 93 ± 5%) rather than by body expressions (87 ± 9%). The ANOVA also showed a significant main effect of the factor Expression (*F*_2,46_ = 5.16; *p* = 0.009; *η*_*p*_^*2*^ = 0.18) that was qualified by the critical Facial Manipulation x Expression interaction (*F*_4,92_ = 2.61; *p* = 0.04; *η*_*p*_^*2*^ = 0.10; Fig 4). We observed no other significant main effects or interactions in the ANOVA (all *F* ≤ 1.58, all *p* ≥ 0.22), including the higher order interaction with the factor Medium (*F*_4,92_ = 0.22, *p* = 0.93), suggesting that the influence exerted by facial manipulation on emotion recognition was comparable for facial and bodily stimuli.

Post hoc analysis of the Facial Manipulation x Expression interaction indicated that, in all the facial manipulation conditions, recognition of happy expressions (range: 85.7–88%) was less accurate than recognition of neutral and fearful expressions (range: 91.3–92.9%; all *p* < 0.001; all Cohen’s *d* ≥ 0.57), which in turn did not differ from one another (all *p* ≥ 0.36). Importantly, recognition of happy expressions was worse in the Bite condition (85.7 ± 10%) than in the Rest (88.0 ± 10%; *p* = 0.03; Cohen’s *d* = 0.55) and Lip conditions (87.4 ± 11%; *p* = 0.051; Cohen’s *d* = 0.30), which in turn did not differ from one another (*p* = 0.52). In contrast, recognition of neutral and fearful expressions was comparable across facial manipulations (all *p* ≥ 0.36).

In sum, biting a pen reduced recognition of happy, but not neutral or fearful, expressions. However, we further interrogated our data to rule out a possible alternative hypothesis. Indeed, our forced choice task implied that, in the Bite condition, participants tended to miss happy expressions to a greater extent than in the two control conditions. In principle, these changes in performance could be due to discomfort experienced by participants asked to keep the pen between their teeth for prolonged periods. This potentially negative state could have encouraged the use of “negative” responses to happy expressions in the Bite condition relative to the other control conditions. To rule out this possibility, we performed a further Facial manipulation x Error type ANOVA on the percentage of erroneous responses to happy expressions, to check whether the Bite condition tended to change the proportion of the erroneous responses “neutral” or “fear” relative to the two control conditions. Overall, participants tended to provide the erroneous response “neutral” to a greater extent than the erroneous response “fear” (10 ± 9% vs. 3 ± 2%; *F*_1,23_ = 14.27, *p* < 0.001; *η*_*p*_^*2*^ = 0.38). However, this tendency was comparable across facial manipulations, as no interaction with that factor was detected (*F*_2,46_ = 0.03, *p* = 0.97).

The analysis of RTs showed that participants responded differently to facial and body emotional expressions (S2 Text). Critically, however, there was no influence of facial manipulation, thus indicating that biting a pen affected response accuracy but not speed.

### Experiment 2

**Fig 5:**
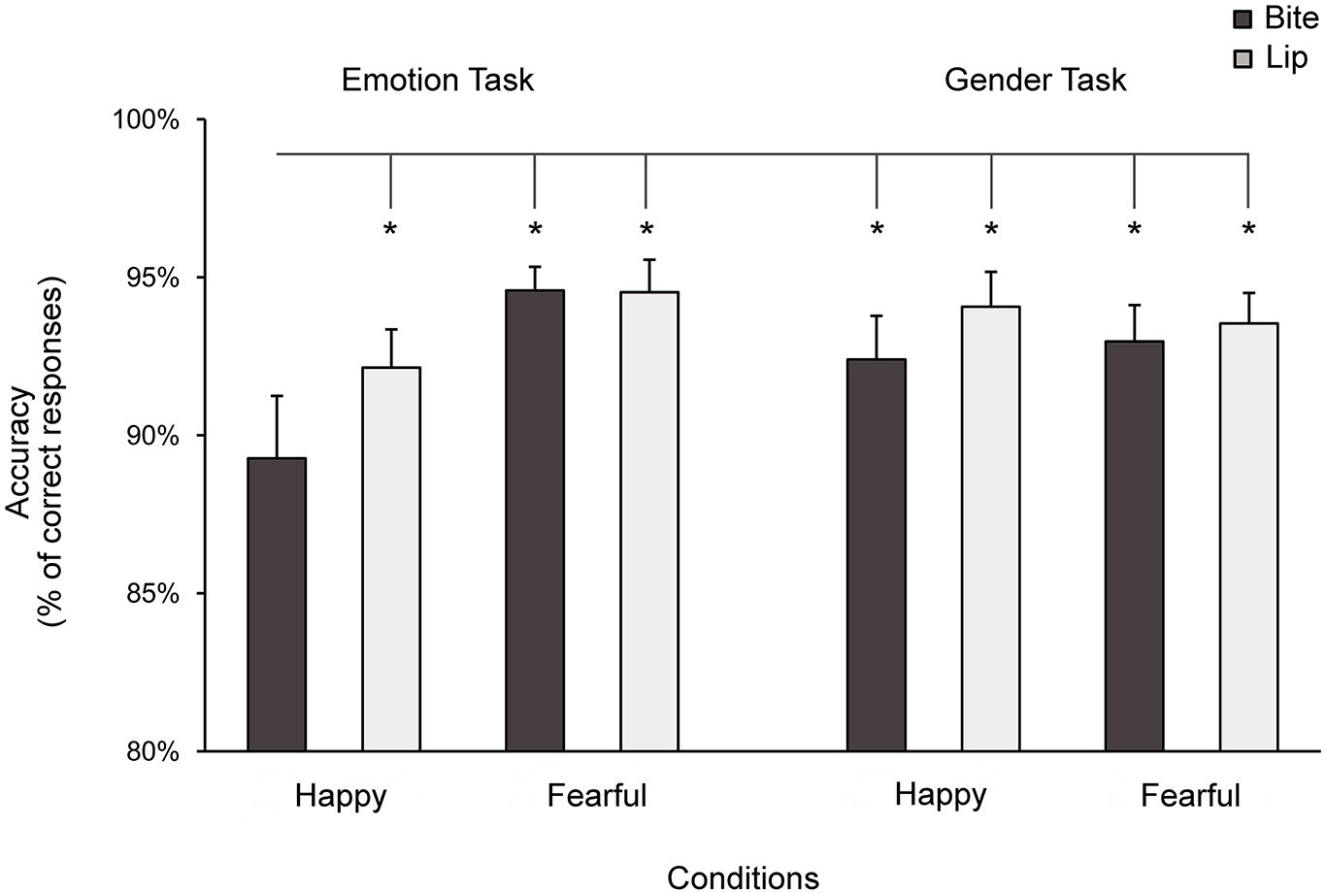
Mean accuracy in Experiment 2. When biting a pen, participants’ ability to accurately identify happy expressions was decreased relative to all the other conditions. Asterisks indicate significant comparisons. Error bars indicate s.e.m.

The Medium x Task x Condition ANOVA carried out on accuracy data showed a significant main effect of the factor Condition (*F*_3,69_ = 4.87; *p* = 0.004; *η*_*p*_^*2*^ = 0.17), which was qualified by a Task x Condition interaction (*F*_3,69_ = 4.07; *p* = 0.01; *η*_*p*_^*2*^ = 0.15; Fig 5). The interaction showed that the reduction in accuracy for happy expressions while biting a pen was specific to the emotion recognition task. Post hoc analysis showed that, during this task, participants’ ability to identify happy expressions while biting a pen (89 ± 10%) was lower than when they had to identify happy expressions while holding the pen with their lips (92 ± 6%, *p* = 0.01, Cohen’s *d* = 0.43) and or when they had to identify fearful expressions while biting a pen (95 ± 4%, *p* < 0.001, Cohen’s *d* = 0.64) or holding it with their lips (95 ± 5%, *p* < 0.001, Cohen’s *d* = 0.58). During the emotion recognition task, we observed no significant differences between the Bite-fearful, Lip-happy and Lip-fearful conditions (all *p* ≥ 0.29). Also, we observed no significant differences between conditions in the gender recognition task (accuracy range: 92–94%; all *p* ≥ 0.46). Lastly, performance in the critical condition, i.e., Bite-happy of the emotion recognition task, was lower than performance in any of the conditions of the gender recognition task (all *p* ≤ 0.02, all Cohen’s *d* ≥ 0.48). As in Experiment 1, the disruptive effect of biting a pen on the recognition of happy expressions was similar across facial and body expressions, as no higher-order Medium x Task x Condition interaction was found (*F*_3,69_ = 0.17; *p* = 0.92).

The ANOVA also showed a Medium x Task interaction (*F*_1,23_ = 32.36; *p* < 0.001; *η*_*p*_^*2*^ = 0.58), indicating that participants were better at discriminating emotions from bodies than from faces (94 ± 5% vs. 92 ± 6%; *p* = 0.01; Cohen’s *d* = 0.67) and better at discriminating gender from faces than from bodies (95 ± 5% vs. 91 ± 6%; *p* < 0.001; Cohen’s *d* = 0.78). The difference between these results and what we observed in Experiment 1 (i.e., greater accuracy at discriminating emotions from faces than from bodies) may be ascribed to physical differences between the stimuli. Faces in Experiment 2 were cropped in an elliptical shape to exclude hair, ears and necks, thus eliminating their potential influence on gender or emotion recognition, and the chest of each body was occluded using a black strip. Alternatively, the difference could be due to the fact that we removed the neutral expressions in Experiment 2, or that participants were asked to perform a gender task, as well.

The Medium x Task interaction effect was similar across the factor Condition, as the 3-way interaction was not significant. No other significant main effects or interactions were observed in the ANOVA (all F ≤ 2.14, all *p* ≥ 0.16).

The analysis of RTs showed that when participants had to discriminate the emotion, they performed better (faster RTs) with bodies than with faces, whereas when they had to discriminate gender, they performed better with faces than with bodies (S2 Text), a trend in line with the accuracy results. Critically, there was no effect of Condition, indicating that biting a pen affected recognition accuracy but not response speed.

### Emotion recognition performance across Experiments 1 and 2

The Experiment x Medium x Facial manipulation x Expression ANOVA showed a main effect of Facial manipulation (*F*_1,46_ = 4.76, *p* = 0.03; *η*_*p*_^*2*^ = 0.09), a significant main effect of Expression (*F*_1,46_ = 22.04, *p* < 0.001; *η*_*p*_^*2*^ = 0.32) and importantly, a significant Facial Manipulation x Expression interaction (*F*_1,46_ = 4.35, *p* = 0.04; *η*_*p*_^*2*^ = 0.09), which confirmed the reduction in happy expression recognition accuracy when biting a pen (88 ± 6%) relative to the other conditions (range: 90–93%; all *p* ≤ 0.002). This effect was comparable across the two experiments and face/body mediums, as we observed no higher-order interactions involving those factors (all *F* ≤ 0.29, all *p* ≥ 0.59).

### Relation between changes in accuracy and dispositional empathy

**Fig 6:**
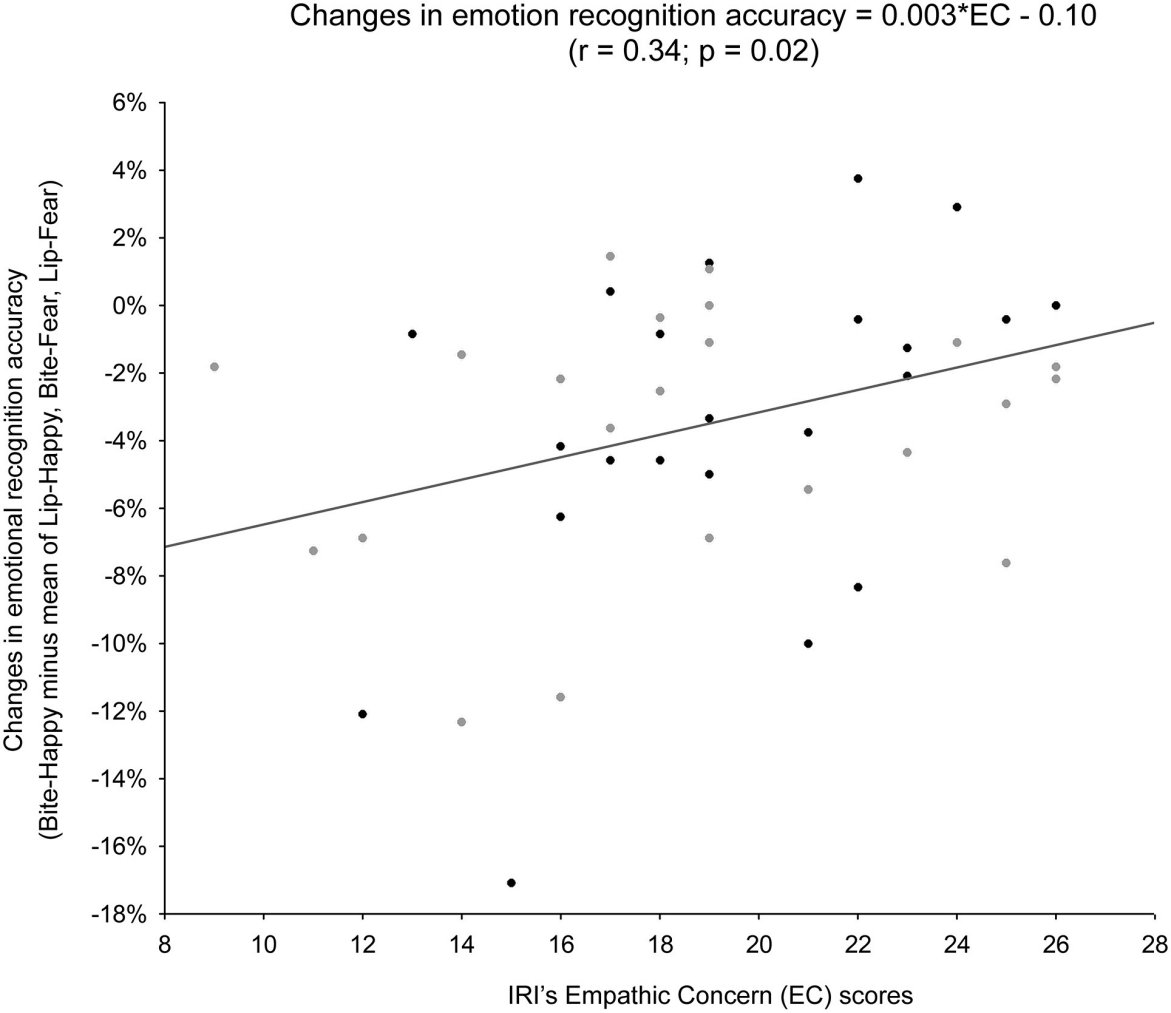
Relation between changes in accuracy and individual differences in empathy disposition. Scatter plot of the correlation between the behavioral index reflecting the costly influence of biting a pen on recognition of happy faces (accuracy in the Bite-happy condition minus mean accuracy across Lip-happy, Bite-fear and Lip-fear conditions) and individual scores on the IRI’s EC subscale (r = 0.34, p = 0.02). Grey and black dots represent participants in Experiment 1 and 2, respectively.

Based on the results of the ANOVAs, we extracted an index of the drop in accuracy reflecting the costly effect of biting a pen on emotion recognition (i.e., accuracy in the Bite-happy condition minus mean accuracy across Lip-happy, Bite-fear and Lip-fear conditions). Negative values of this behavioral index indicate greater interference. We tested whether inter-individual differences in dispositional empathy predict that index across the two experiments. The regression model with the IRI subscales, Experiment and their interactions as predictors was not significant (*R*^*2*^ = 0.11; *F*_9,38_ = 0.50, *p* = 0.87), and showed a non-significant improvement after the removal of two outliers with standard residuals >2.5σ (*R*^*2*^ = 0.19; *F*_9,36_ = 0.92, *p* = 0.52). The EC subscale was the only significant predictor of the behavioral index (*β* = 0.50, *F*_1,39_ = 5.49, *p* = 0.02, *ηp*^*2*^ = 0.13); there was no relation between the bite-related drop in accuracy and the other IRI subscales, the factor Experiment or any interactions between IRI subscales and Experiment (all *F* ≤ 2.77, all *p* ≥ 0.11). To further check whether the influence of EC on the behavioral index was comparable in the two experiments, a further regression model with the factor EC, Experiment, and their interaction was performed. The model was marginally significant (*R*^*2*^ = 0.16; *F*_3,42_ = 2.76, *p* = 0.054, *f*^*2*^ = 0.20) and the factor EC remained a significant predictor of the behavioral index (*β* = 0.40, *F*_1,42_ = 7.49, *p* = 0.009, *η*_*p*_^*2*^ = 0.15). Again, we observed no influence of the predictor Experiment or the interaction Experiment x EC (all *F* ≤ 2.61, all *p* ≥ 0.11; Fig 6). These findings indicate that dispositional emotional empathy similarly influenced performance in the emotion recognition task across the two experiments. Specifically, the positive relationship between the behavioral index and the IRI’s EC subscale indicates that participants with lower levels of emotional empathy were highly affected by biting a pen, whereas participants with higher levels of emotional empathy showed little or no interference.

Simple correlations computed across the two experiments confirmed that behavioral interference was associated with individual differences in EC scores (*r* = 0.34; *p* = 0.02, *f*^*2*^ = 0.13), but not FS (*r* = 0.14; *p* = 0.37), PT (*r* = 0.13; *p* = 0.39) or PD scores (*r* = –0.09; *p* = 0.57).

## Discussion

Based on previous experimental evidence for the role of facial mimicry in the recognition of others’ emotions, and in the light of the (sensorimotor) simulation models previously proposed, the main purpose of this work was to investigate whether facial mimicry plays a critical role not only in recognizing facial expressions of emotion, but also in recognizing bodily expressions of emotions. Our finding that interfering with facial mimicry not only affected the recognition of emotions expressed through others’ faces but also emotions expressed through others’ bodies supports a simulation model in which facial feedback has a critical role in modulating sensorimotor simulation of a rich expressive motor program (i.e., not only the facial component) associated with the observed emotion. This evidence suggests that the simulation is not limited to the available visual information (i.e., the isolated part of the expression that is visible to the observer), but extends to a whole, rich sensorimotor program associated with the perceived emotion. Remarkably, this evidence demonstrates the functional relevance of these sensorimotor processes to visual emotion recognition.

Our design also allowed us to expand on prior work by comparing recognition of happy expressions with recognition of expressions that have not been consistently tested (e.g., neutral expressions) or have brought less consistent results (e.g., fearful expressions), and by testing the moderating role of dispositional empathy.

In order to test our hypotheses, we implemented two experiments that involved manipulating facial mimicry during visual recognition of emotions displayed by facial and body expressions (i.e., happiness, fear and a neutral state). In Experiment 2, we included a control task in which participants had to recognize the gender (rather than the emotion) of the actor in order to provide clear-cut evidence that interfering with facial mimicry has a selective impact on emotion recognition. The results of both experiments unambiguously demonstrated that manipulation of facial mimicry (in particular, the "Bite" condition specifically implemented to interfere with the activation of facial muscles involved in smiling) affected the recognition of happy expressions, while the recognition of neutral (Experiment 1) and fearful (Experiment 1 and Experiment 2) expressions was not disturbed by the mimicry manipulation. These findings provide evidence in line with studies that demonstrated a correspondence between blocking specific facial muscles and the resulting impairments in recognizing selected facial expressions involving the same muscles [48,49,56,57].

However, as we discuss in the following paragraph, this selectivity does not take for granted that the mechanism underlying facial mimicry is based on a one-to-one match between the observed visual display and the (en)acted facial expression. Indeed, and crucially for the purpose of the present investigation, the “Bite” condition impaired the recognition of happy expressions conveyed by both faces and bodies, thus suggesting that altering facial mimicry interfered with a global sensorimotor simulation of the emotion of happiness. This result provides even clearer evidence when considering that the manipulation of facial mimicry did not affect the recognition of the actors’ gender, revealing a selective role of facial mimicry in the recognition of others’ emotions.

Overall, our findings speak against simulation models in which facial mimicry is considered irrelevant to emotion recognition. Moreover, they do not support a simulation process based on a one-to-one visuo-motor matching mechanism. Rather, our results, in the first instance, seem to support the thesis that what is simulated is a rich (sensori)motor program associated with an emotion. From this perspective, interfering with a component of the (sensori)motor program (i.e., a facial expression of happiness) could affect the whole program—and, as a consequence, it could also interfere with visual recognition of the associated facial and body expressions (of happiness).

Our findings are in keeping with the correlational evidence that emotionally congruent facial EMG responses also occur when seeing emotional expressions conveyed solely by the body [69–72] or the eye regions [87] in the absence of facial visual cues, and with the study of Sel and collegues [88] showing similar widespread recruitment of facial and body neural representations within the somatosensory cortex during discrimination of emotional faces, but not during gender discrimination. Importantly, our findings expand these previous results by demonstrating a functional relevance of these sensorimotor processes to visual recognition of emotional expressions.

Our findings could be explained in the light of a recent sensorimotor simulation model proposed by Wood and colleagues [31]. This model considers facial mimicry as a spill-over of the sensorimotor simulation, but at the same time able to influence, through feedback to the (pre)motor and somatosensory regions, the simulation itself. The model proposes that the observer uses his/her own sensorimotor system to simulate the observed emotional expression, which would then be followed by multiple, cascading activations of a series of other systems, including the limbic system and regions mostly involved in reasoning about others’ mental and affective states. Crucially, the model also establishes that feedback from facial mimicry may shape the entire activated emotional state by means of connections between regions devoted to sensorimotor simulation and regions associated with the processing of emotions and the ability to reason about others’ mental and affective states.

Notably, Wood and colleagues [31] proposed the existence of a direct path from the visual system to the emotional system. The recruitment of the sensorimotor system, from this perspective, could also follow (rather than precede) the activation of the emotional system. This is in keeping with scholars who proposed that facial mimicry could reflect the activation of an affective state in the observer rather than a one-to-one visuo-motor matching response [69–72,87]. Studies have also supported the proposal that observers “mimic” their interpretation of the observed expression rather than the specific motor patterns observed [71,89,90]. Taken together, these lines of evidence suggest an alternative pathway to facial mimicry which follows the activation of the emotional system and, likely, of other systems responsible for reasoning about others’ mental and affective states. Therefore, facial mimicry can be the result of top-down processes in addition to the result of a bottom-up mechanism linked with the perception-action loop.

It should be noted however, that sensorimotor simulation could occur at different levels of complexity, as documented by action perception literature. While seeing an (emotionally neutral) action facilitates corresponding motor circuits for making the same observed action, thus supporting one-to-one matching mechanisms [91–93], studies have shown that such a facilitation occurs even when the observed action is occluded from view and can be only inferred based on contextual cues [94,95]. Moreover, when an individual observes a goal-directed action, the motor system can encode the distal goal of the action rather than the specific movements being observed [96–98]. These findings have been interpreted either in terms of learned associations between multiple action cues that are encoded within the sensorimotor system [e.g., 95,99] or top-down influences from other brain systems (e.g., semantic or theory of mind systems) [100,101].

From this perspective, seeing a happy body expression could induce a sensorimotor simulation of the whole sensorimotor program associated with happiness (including happy facial expressions) either based on learned associations between happy bodily and facial cues or via top-down influences from other systems involved in processing social and emotional signals (e.g., emotion/theory of mind systems). Our findings, although intriguing, do not allow us to disentangle between these two different possible mechanisms for facial mimicry—one directly mediated by the visual input *via* sensorimotor simulation and one mediated by the emotion/theory of mind systems *via* sensorimotor simulation—and investigations aimed at clarifying the time course and computational steps involved in the entire process are required. Our view, which also seems to be embraced in the recent model by Wood and colleagues [31], is that facial mimicry can result from an interplay between simulative bottom-up processes and top-down processes, for example, linked to the observer’s interpretation of the other person’s expression.

We also observed a relationship between individual levels of empathy and performance impairment in the emotion discrimination task, such that the participants with higher/lower scores on the IRI EC subscale were those who showed a lesser/greater impairment due to the mimicry manipulation. On the other hand, we found no relationship between behavioral interference and cognitive subscales of the IRI. Cognitive and emotional empathy correspond to the abilities to cognitively understand and affectively share what others think and feel. In particular, the IRI’s EC scale assesses the tendency to experience other-oriented feelings such as sympathy and compassion which do not involve affect sharing and “feeling as” the other, but rather a “feeling for” and thus concern and attention to others and their needs [75]. Neuroscientific studies have linked this empathic disposition with brain regions associated with affective responses, reward processing, action and cognition, such as the anterior insula, anterior/subgenual cingulate and inferior frontal cortex [102–106]. Interestingly, previous studies have consistently documented that people scoring high on EC or similar emotional empathy scales (rather than on cognitive empathy scales) display greater emotionally congruent facial mimicry when exposed to emotional faces [14,16,18,74], possibly because of their greater motivation to attend to others’ emotional signals [75]. However, the correlational nature of these prior studies did not allow them to determine whether participants with high emotional empathy also *required* facial mimicry to a greater extent in order to accurately recognize others’ emotional expressions.

In striking contrast with this possibility, our study suggests that participants scoring high at the IRI’s EC subscale can rely on facial mimicry to a lesser extent in order to achieve accurate emotion recognition, as these participants were less affected by mimicry blocking. Although highly empathic individuals were reported to display greater spontaneous facial mimicry [14,16,18,74], the processing underlying facial mimicry appears to be less functionally relevant for achieving accurate emotion recognition in these individuals. This does not imply that mimicry blocking is simply not effective in these participants. Indeed, a previous study [53] reported that people scoring high on a global scale of empathy (involving not only emotional but also cognitive empathy traits) displayed greater effects of mimicry blocking on brain responses to emotional faces (i.e., on an electro-cortical component of event-related potentials thought to reflect the quality of visual working memory representation). Rather, in light of this prior work, our findings suggest that highly empathic participants tend to compensate for interference with facial mimicry by using alternative pathways that do not rely on sensorimotor simulation, such as from visual to emotional and theory of mind systems.

In keeping with this proposal, a recent TMS study investigated the causal role of sensorimotor and mentalizing brain networks in understanding others’ emotional feelings when watching smiling expressions, and the moderating role of inter-individual differences in empathy [43]. In line with our behavioral results, that study found that more empathic participants were less disrupted by rTMS over the face representation in sensorimotor brain regions. On the other hand, they showed impaired performance only when the TPJ (a key area in the theory of mind system) was targeted. All in all, these studies indicate that more empathic individuals tend to spontaneously show behavioral markers of sensorimotor simulation, which may reflect greater attention to others’ emotional signals, but at the same time (and probably because of such greater attention) they are able to compensate for interference with sensorimotor simulation by using other cognitive/neural resources.

A limitation of our study is the sample size, which appears smaller than in some previous studies that tested the effect of mimicry blocking on emotion recognition [49,56,57]. Yet, to implement the mimicry manipulation, we used a repeated measures design which is more sensitive to detecting significant differences. Moreover, potential concerns of data replicability (e.g., [107]) are at least partially attenuated by the consideration that the effect of mimicry blocking on recognition of happy expressions—at least facial expressions—is consistent with previous reports [48,49,56,57], and here it was replicated across the two experiments. Future studies with larger sample sizes may seek to confirm the present findings.

## Supporting information

S1 Text Pilot study 1 and 2. Description of the procedures used to select visual stimuli for Experiment 1 and 2.
(DOCX)

S2 Text Analysis of RTs in Experiment 1 and 2. Description of the statistical analyses performed on RTs data in the main experiments.
(DOCX)

S1 Table Mean ratings of stimuli selected in pilot study 1. Mean ± S.D. ratings of happiness and fear reported on a 9-point Likert scale ranging from 1 (no emotion) to 9 (maximal intensity of the emotion).
(TIF)

S2 Table Mean recognition accuracy of stimuli selected in pilot study 2. Mean ± S.D. of emotion and gender recognition accuracy (% of correct response).
(TIF)
