# Supplementary material for: Blocking facial mimicry affects recognition of facial and body expressions
Source: PLoS One. 2020 Feb 20;15(2):e0229364. doi: 10.1371/journal.pone.0229364 (PMC7032686; doi:10.1371/journal.pone.0229364)
Supplement: S1 Text — Description of the procedures used to select visual stimuli for Experiment 1 and 2. (DOCX) [file pone.0229364.s001.docx]

**Pilot study 1: selection of visual stimuli for Experiment 1**

In the first pilot study, 20 participants were presented with an initial pool of 214 body expressions taken from a validated database showing emotional and neutral expressions of the bodies of male actors whose faces were blanked out [58,76]. Moreover, participants were presented with 158 facial expressions that were created by modifying stimuli from the Nimstim database [77] in order to start with a sample of facial expressions showing different degrees of emotional intensity. For each actor, we created morphing transitions between the neutral face and the final expressive face using Fantamorph software ([http://www.fantamorph.com](http://www.fantamorph.com/)). Participants were presented with the 372 pictures and asked to judge the intensity of happiness and fear conveyed by each expression using a 9-point Likert scale ranging from 1 (no emotion) to 9 (maximal intensity of the emotion). To avoid building up artificial correlations between the different evaluations, each rating was collected separately during successive presentations of the whole set of stimuli. Facial and body stimuli were presented in separate blocks. Stimuli remained on the screen until the participants’ response. Ratings were self-paced and we did not collect RTs. The order of ratings, blocks and stimuli was randomized. Based on the mean intensity ratings of each stimulus across the 20 participants, we selected three groups of emotional expressions for each face/body medium: happy expressions (mean ratings of happiness: 5 ≥ h ≥ 8; mean ratings of fear: f ≤ 3), fearful expressions (mean ratings of fear: 5 ≥ f ≥ 8; mean ratings of happiness: h ≤ 3) and neutral expressions (mean ratings of happiness and fear: h and f ≤ 3). This procedure ensured that the selected expressions conveyed appropriate emotional information. In this way, we also discarded emotional stimuli with extremely high intensity ratings, to ensure recognition of the stimuli was not trivial. Then, an experimenter further selected 138 stimuli (23 stimuli for each combination of face/body medium and emotional expression), with the aim of achieving a balanced representation of 4 actors while maintaining similar ratings between facial and body stimuli. S1 Table shows subjective ratings of the final set of stimuli.

**Pilot study 2: selection of visual stimuli for Experiment 2**

In the second pilot study, participants were presented with an initial pool of stimuli belonging to the same databases used in Experiment 1 [58,76,77]. The initial pool comprised 138 body stimuli and 140 face stimuli, equally distributed across four stimulus categories, namely happy females, happy males, fearful females and fearful males. Participants performed two tasks presented in two separate sessions, whose order was counterbalanced across participants. In each task/session, participants were presented with 5 blocks during which the whole set of stimuli was presented on the screen in a randomized order. They were asked to report either the gender (gender discrimination task) or the emotion expressed by the model (emotion discrimination task). Each picture was presented for 500 ms and the order of the tasks and blocks was randomized. Based on participants’ performance, we selected a pool of 40 faces and 40 bodies.
