# Supplementary material for: Blocking facial mimicry affects recognition of facial and body expressions
Source: PLoS One. 2020 Feb 20;15(2):e0229364. doi: 10.1371/journal.pone.0229364 (PMC7032686; doi:10.1371/journal.pone.0229364)
Supplement: S2 Text — Description of the statistical analyses performed on RTs data in the main experiments. (DOCX) [file pone.0229364.s002.docx]

**Analysis of RTs data in Experiment 1.**

In Experiment 1, the Medium x Facial Manipulation x Expression ANOVA carried out on RTs showed a main effect of the factor Expression (*F*_2,46_ = 4.69; *p* = 0.01; *η_p_*^2^ = 0.17) and a Medium x Expression interaction (*F*_2,46_ = 16.78; *p* < 0.001; *η_p_*^2^ = 0.42). For facial stimuli, RTs to neutral expressions (660 ± 109 ms) were faster than RTs to happy expressions (711 ± 111 ms; *p* < 0.001; Cohen’s *d* = 0.80) and fearful expressions (717 ± 115 ms; *p* < 0.001; Cohen’s *d* = 0.94), which in turn did not differ from one another (*p* = 0.91). In contrast, for body stimuli, RTs to fearful expressions (673 ± 105 ms) were faster compared to happy expressions (716 ± 126 ms; *p* = 0.003; Cohen’s *d* = 0.82) and neutral expressions (712 ± 127 ms; *p* = 0.004; Cohen’s *d* = 0.60), which in turn did not differ from one another (*p* = 0.89). These findings can be accounted for by the visual distinctiveness of our pool of facial and body stimuli: fearful bodies were the only ones showing crouched postures and neutral faces were the only ones showing a static/relaxed face. These features could have made recognition of those stimuli faster relative to the other facial and body categories. No other main effects or interactions were significant (all *F* ≤ 1.81, all *p* ≥ 0.17), including the Facial manipulation x Expression interaction (*F*_4,92_ = 0.28; *p* = 0.89). Thus, the reduction in accuracy observed in Experiment 1 for happy expressions when participants bit a pen cannot be due to any speed-accuracy trade-off.

**Analysis of RTs data in Experiment 2.**

In Experiment 2, the Medium x Task x Condition ANOVA carried out on RTs showed a significant main effect of the factor Medium (*F*_1,23_ = 6.77; *p* = 0.02; *η_p_*^2^ = 0.23), which was qualified by a Medium x Task interaction (*F*_1,23_ = 53.85; *p* < 0.0001; *η_p_*^2^ = 0.70). This interaction showed that participants were faster at discriminating emotions from bodies than from faces (551 ± 95 ms vs. 575 ± 112 ms; *p* = 0.02; Cohen’s *d* = 0.41) and faster at discriminating gender from faces than from bodies (543 ± 105 ms vs. 568 ± 112 ms; *p* < 0.001; Cohen’s *d* = 0.50). No other main effects or interactions approached significance (all *F* ≤ 3.22, all *p* ≥ 0.08), including the critical Task x Condition interaction (*F*_3,693_ = 0.19; *p* = 0.90). Thus, the reduction in accuracy observed in Experiment 2 for happy expressions when participants bit a pen cannot be due to any speed-accuracy trade-off.
