# Supplementary figures and images for: Blocking facial mimicry affects recognition of facial and body expressions
Source: PLoS One. 2020 Feb 20;15(2):e0229364. doi: 10.1371/journal.pone.0229364 (PMC7032686; doi:10.1371/journal.pone.0229364)

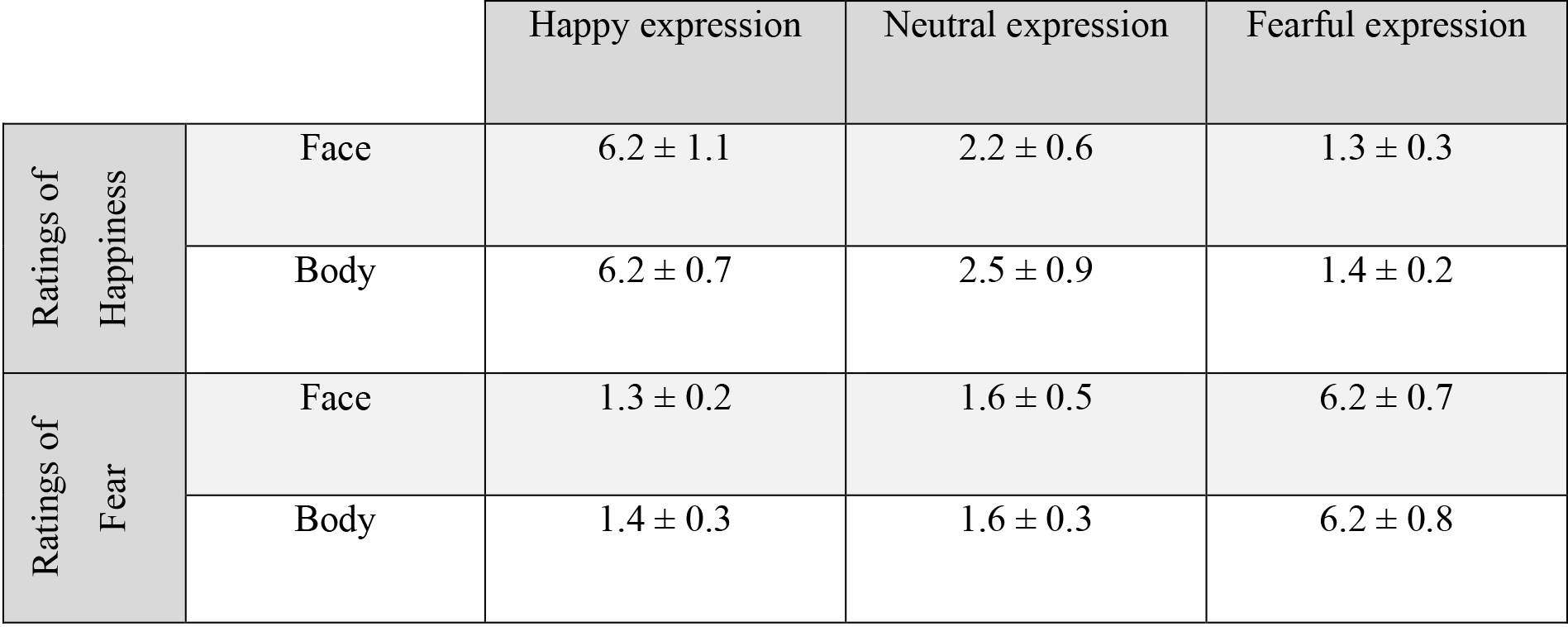

Supplement: S1 Table — Mean ± S.D. ratings of happiness and fear reported on a 9-point Likert scale ranging from 1 (no emotion) to 9 (maximal intensity of the emotion). (TIF) [file pone.0229364.s003.tif]

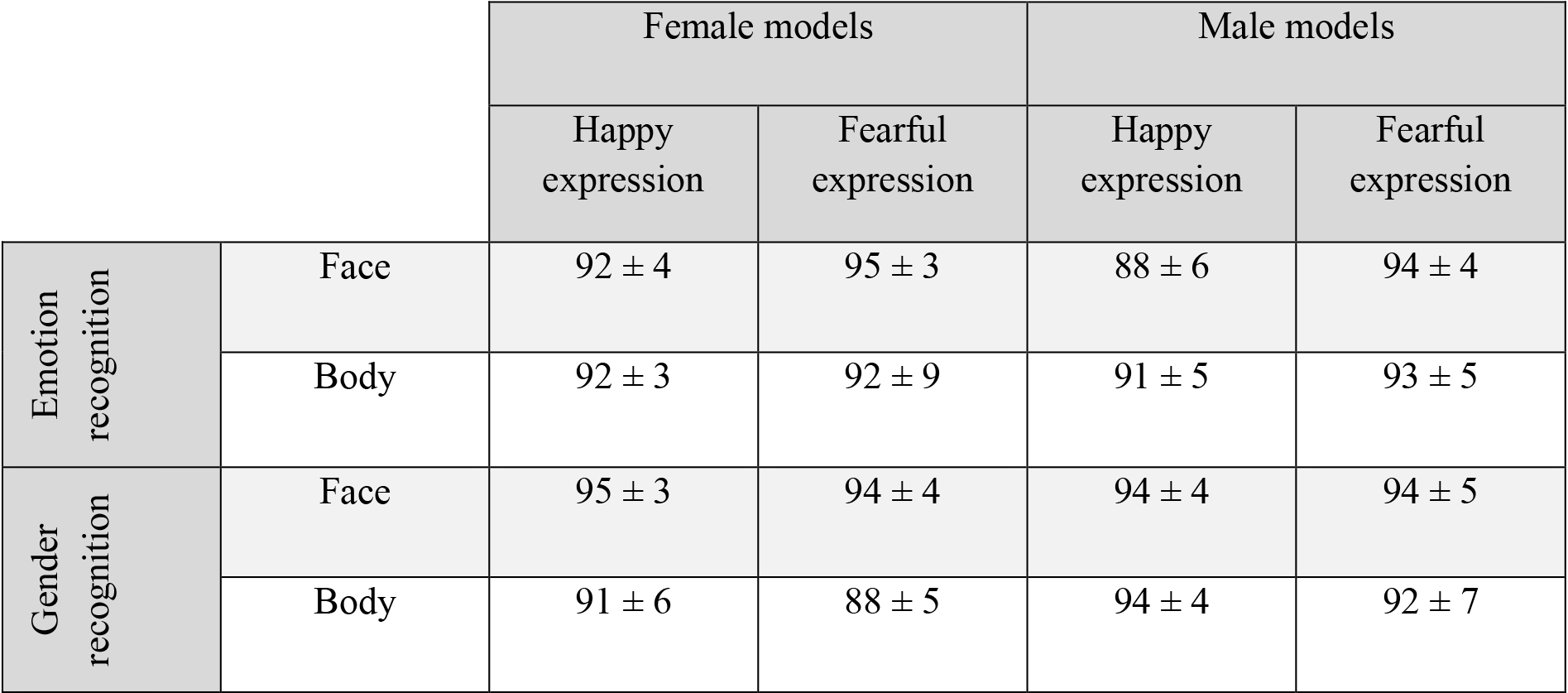

Supplement: S2 Table — Mean ± S.D. of emotion and gender recognition accuracy (% of correct response). (TIF) [file pone.0229364.s004.tif]
